# Advantages of Combination in Therapeutics

**Published:** 1869-06

**Authors:** David E. Chace


BUFFALO

### Medical and Surgical Journal.

VOL. VIII. JUNE, 1869. No. 11.

### Original Communications.

ART. I.—*Advantages of Combination in Therapeutics.* BY DAVID E. CHACE, M. D. *Read before the Erie County Medical Society, June* 8, 1869.

Physiological research and experimentation united to pathological investigation have, of late years, materially altered the views entertained by scientific men in regard to the vexed question, diseases and their remedies. To one, who, with careful thought, peruses the pages of historic medicine, it appears as if one stood upon the threshold of a grand and glorious future. An age characterized by one essential feature, progress, in all that pertains to science and to art, must not forsooth be barren in that, which we, I trust, deem as the object of our lives, the advancement of the healing art. The means by which this is to be accomplished have never before been perfectly placed at our disposal. Nay, more, physiology is but of late taught as it should be, and pathology too, has been a secondary consideration until within comparatively a few years. Yet even now, there are many serious obstacles to be overcome ere science shall issue forth from the contest triumphant, and truth be vindicated. For many are there who by reason of long entertained opinions, convictions if you will, or still worse, those who, grasping an idea unaided, have deemed it a wonderful discovery, and made facts and converts bend to their imperious diction, alike retard progression. Prejudice is a terrible opponent, and be it induced by idiosyncrasy of individual or by misfortune of birthplace, it is none the less hurtful or injurious. What we desire in medicine are facts, and come these from whatever nation or people outside our own, let us be men enough to acknowledge their merit. To-day I enjoy the privilege of presenting you thoughts culled from various soils, and though these shall appear uninteresting or unprofitable, my inexperience will, I trust, be my apology. In tracing a new path, I am aware of the difficulties, and led perchance, rather by ambition than by discretion, I have attempted to accomplish too great an undertaking. Be that as it may, should I add one iota to the cause of relief to suffering humanity, I am content.

To the thorough and comprehensive understanding of our subject, it is necessary that we view first, the condition disease, and next the means by which its alleviation, if effected, viz.: remedies. In this paper, general ideas only can be advanced, and certain facts being taken as premises, we shall thence strive to logically deduct conclusions. Disease consists in states induced by the violations of nature's laws, or by an obstruction so situate that the normal function of a part or parts of the human system cannot be performed harmoniously. Taking as a departure, physiological truths, we notice the intimate relation of the various portions of the human body, as well as the fact that when once the balance is never so slightly misplaced, all portions are made to comprehend the inaccuracy. In the nerves system—in those ganglia about which we read so much and know so little, is held the key with which to unlock the hidden mysteries. As yet we have learned but the alphabet of nervous action, and when if ever its language shall be spoken, the clouds that now envelop us will disappear and light may dawn forever.

To resume. This net-work of fibres, embracing every organ, penetrating every tissue, is the medium by which diseased action is made to extend as if by sympathy to portions the most remote. Again, disease once established, passes through a series of successive changes to a recovery or to dissolution. If the former, it necessarily leaves that part or that organ with a lesion, be it of structural change or of perverted function. The increase of disorders hereditary in their nature, the taint of phthisis or the curse of syphilis, together with the malarious and other miasmatic influences brought to bear upon the human family, have, or ought to have, in my judgment, given a new aspect to our therapeutics, a branch of medicine that has of late been, in the opinion of those high in authority, but too little studied. That there is a reason for this omission is obvious to all who reflect. A new remedy is brought forward—is tried—and with success. Forthwith a series of experimentations are made which would be sufficient were the results all satisfactory, to give it preëminence above all other remedies, by reason of its supposed virtues. Time, the great test, however, finds it wanting in most cases, and the physician casts it aside, having tried it in diseases for which it was not originally designed, or by an unfortunate choice prescribed it in but a few isolated or complicated disorders. This method of investigation certainly tends to destroy confidence in medicines and in their powers. Justice claims that fair opportunity and careful procedure should mark the trial of the new candidate for the alleviation of misery.

The rule laid down by authors is, that simples should be used as often as possible, but most of these further state, that a judicious combination of remedies is often far more efficient. The observation of those whose experience and judgment are to be relied upon, and to whom moreover the younger members of the profession are to look for advice, will, I think, bear testimony in favor of this statement. The growing distrust in remedies, manifested by many members of the profession is, to say the least, unfortunate, more especially as it occurs at a time when quackery has unbridled license (in the form of authority) to pay homage to the follies of the people, and triumph over rational and scientific medicine. These gentlemen while devoting their efforts, and with all honesty of purpose I have not the slightest hesitation in asserting, to the cure of affections by simple remedies, do not, I imagine show, other things being equal, a greater number of cures in a given number of instances. When they can do this, or when a specific treatment shall be found for all disorders, it will be just to give credence to their theories. Our great danger is that we are unable to preserve a medium between the two extremes.

Many of you, gentlemen, can recall the period of universal blood-letting, of the inordinate use of mercury and kindred articles of the materia medica, and to-day we are so far reformed that we must forsooth discard these in toto. Because they have been used in excess they must not be employed with toleration in proper proportions and in proper subjects. As it is not true that since many disorders terminate in restoration of health without medicine, we should never employ any in these affections, so it is equally false that symptoms varied in their nature, pointing to as varied complications, can be relieved by entire simplicity of prescription.

The preëminent importance attached to therapeutics, is founded upon the fact that the grand object of medicine, as a science, is to remove disease. In order to accomplish this, the indication necessarily is to restore order to organic functions, and thus to remove any alteration in tissue that may be the consequence of such disordered functions. The precise nature of the disease—the part especially affected is to be discovered by a diagnosis founded upon a knowledge of the healthy functions and condition, as also of the morbid appearances which follow as the results of perverted function or nutrition. This attained, we turn to therapeutical agencies to fulfill the indications presented us by such an investigation. Nor are these to be treated simply, for far beyond this we have the cause of which these are but the results. Reason and judgment combined having found the one, the selection of medicinal agents and the mode of action becomes at once simplified, though perchance our expectations are not at times as fully realized as we could desire. What then are we to gain by a combination of remedies? How are we to know which of the agents employed is chiefly instrumental in producing a cure? Taking the old form of prescription we have the basis—the principal agent which we are aware will, under certain circumstances, produce a given effect, together with this we give others which, as adjuncts, modify or increase the action of such and such an article of the materia medica. Experience is the only criterion afforded us, and the observations made on a large scale in institutions governed by men of acknowledged ability and integrity, are the witnesses which certify as strongly as possible to the truth of such statements. To those who cavil at facts as well as men, nothing can be adduced.

For medicine is not, as I am aware, a science, demonstrable like mathematics, wherein certain truths cannot be gainsayed or disproven.

I. "The basis, then, being united with a substance or substances of like nature, but of less efficacy or energy (individually) in their operation the desired action is materially increased." As this is daily brought into the notice of every practitioner I need only call your attention to a little point demonstrated lately in England, viz.: in regard to the combination of narcotics for the relief of pain. Instances are cited in which neither morphia, opium or belladonna given singly, in reasonable doses, would attain the object, but when two of these, say belladonna and morphia, were combined, pain was allayed, though sleep did not always follow. This is not, perhaps, a new feature, for there are in reality but very few *new* ideas advanced which, if they possess merit, do not have their foundation in something dating back a century or two. Personally I am satisfied that such results do obtain, and for the sake of illustration would mention a case of a lady, a sufferer from an aggravated and painful attack of disease, to whom acet. of morphia was faithfully administered and in comparatively large doses, who was finally relieved by the administration of a small quantity of belladonna and the above mentioned salt. It were almost unnecessary for me to state how jalap and colocynth and rheubarb are rendered more efficient by calomel, or that diaphoresis is more readily produced by opium and ipicachuana, than by either singly, or that digitalis as a diuretic is not always certain unless combined with squills or some kindred article.

II. Again: "The basis may be modified in its action or relieved of some of its otherwise unpleasant effects by judicious combination." Given in quantities sufficient to accomplish desired results, medicines, such as those of the diuretic or cathartic orders, by persistent use, lose their power, and as certain chronic cases require a long continuance of such agents, means must be found to preserve their proper and full effects. Hence aloes in constipation is rendered doubly useful by the addition of hyoscyamus and nux vomica, the one to obviate the pain and griping, the other to give tone to a tract whose nervous energy has been relaxed by reason of impaction within the canal. Sulphate of magnesia is corrected in its tendency to gripe by sulphuric acid in excess. Elaterium in dropsy, together with ext. hyoscyamus, makes a convenient and easily taken purgative. Croton oil or turpentine are disguised by being given in a small amount of castor oil. To these may be added the various methods of preserving intact the stomach, by the employment of otherwise deleterious substances through a mucilaginous medium.

III. "Two substances employed for different purposes, and different eonsequently in their nature, can be used with advantage." Perhaps no one remedy has proven this proposition more effectually than iron. Of late we find it used daily, not always it may be with discretion, but certainly with no sparing hand. Quinine, too, has taken the foremost rank, and the records of prescriptions kept by druggists, show the preponderance of these two remedies. The varied preparations of the above mentioned articles, and the comparative facility with which they can be added to powder, pill or mixture, render these invaluable. Hence tonics are administered with purgatives in cases of anæmia, attended with constipation. Astringents with tonics in those in whom an hemorrhagic diathesis is apparent or suspected. Astringents with narcotics in diarrhœa, attended with much irritability of intestine. Purgatives may be combined with tonics, as in dropsy, where the indication is to remove the fluid, as also to support a weakened constitution. Lastly, antispasmodics are advantageously combined with tonic preparations in patients with disordered nervous systems and an anæmic habit. As vehicles the bitter infusions afford us a convenient method of prescribing a tonic; and these may be added to most mixtures, and doubtless with efficacy. For instance a person who, at a remote period, was subject to malarious influence, or who may be surrounded by it, needs at this time a remedy, say iodide of potassium for a particular condition. This agent can be readily given in an infusion of cinchona, and as far as the prescription is concerned, it is rational.

IV. "By combination we may obtain a new substance different from those used in its formation, and one of increased efficacy." To the skeptical this idea affords the greatest reason for opposition, and could we not point to such long established compounds as Dover's powder and the compound calomel pill of the pharmacopia, we might hesitate to affirm the above. Iodide of potassium with carbonate of ammonia gives rise to the iodide of ammonium, a valuable agent in croupal conditions. Bi carbonate of potassa and iodide of potassium as employed by Dr. Thos. King Chambers, have proven themselves of no mean advantage in rheumatism, to which may be united tinct. of cimicifuga, and in those of gouty diathesis the wine of colchicum.

Moreover, many practitioners deem it best in syphilitic cures to use the bi chloride of mercury in conjunction with iodide of potassium, thus producing a compound which has the merit of being exactly as far as the mercury is concerned at least, in proportion to the wishes of the prescriber. Many articles chemically incompatible, are in daily use, under the forms laid down by the dispensatories. To some this incompatibility of some medicines is a pretext for their exceeding caution in prescribing, and herein lies the secret of the advocacy of simples. Either this or a limited knowledge of the laws of affinity and relation between different chemical agents, must be assigned as the foundation for a denial in toto to combination. Nor am I, I think, too severe. Let us see. There are no men received as authorities by the medical profession who advocate the course of these gentlemen. But I may be answered, this is but a new phase of progress which the this age is calculated to develop! This may be. But as the truths of history, as related by the historian, are rendered more conclusive by the testimony of cotemporaries, so also are the virtues of medicine testified to by men of unquestioned veracity, as far back in the dim ages of yore as even the most profound student of the history of medicine will care to search. To those whom the *vis medicatrix naturæ* is all sufficient, it may be well to remark, that at least it is a very pretty and reasonable fiction to believe that the same power which permits morbific influences to exercise sway over humanity, has given to a body of men devoted to their calling, agents taken if you will, from the great storehouse of nature, but by them, and through them, the means of obviating disease and restoring health.

One point remains to be considered, and did I not deem that it avails more than we give it credit for in the grand process of recovery, I should hesitate before bringing it to your attention.

*Rest.* In this one word the materia medica has an auxiliary powerful, yet not pretentious, available at all times, yet never incommodious. Nature is the teacher; 'tis she who presents us the rules for guidance, the weapons too with which to wage our warfare. 'Tis she above all others, who daily points out her method of procedure, and fortunate are those who are willing votaries at her shrine. Nature, however, is not always adequate, nor in all subjects to eliminate disease, and even if she were, an extended period of time, precious to most of us, would be required to bring about the desired end. Just at this moment art assumes control, and guiding, aiding and restoring the latent energies, together they succeed. Pain is an evidence of the necessity of rest. The rheumatic as fully knows this as he who, suffering from peritonitis, adjusts his limbs to the most comfortable position. Man may, by force of will, bear the torture and thwart the intentions of his benefactor, but the process of recovery being interfered with, it assumes a different phase and terminates with lesions never to be eradicated or in dissolution. Obedience is demanded, and unless given, retribution is sure to follow. In the process of recovery too there is a call upon the vitality of the individual. Days, weeks, nay, even months, must be bridged over, and if during this period this strength is to be consumed by daily exertion, there is just so much less remaining to sustain an enfeebled system. The process of waste and repair in a healthy individual should compensate each other, but in illness we have added the demand for more help than can be afforded, if all the organs of the body are kept permanently in action. In functional disorders we must certainly allow time and opportunity for restoration, or else we labor under the disadvantage of disturbing the machinery of the whole human organism. The importance of rest is recognized by all who study the characters of disease, both surgical and medical, and few men, I trust, would even in medical cares, hesitate to avail themselves of an aid which the surgeon deems so important.

In conclusion allow me to express the hope that time, as it bears us onward, will award to us in our day, some of the discoveries which science seems prepared to reveal, that the realization of long wished for knowledge may be granted those than whom none have ever been more diligent or faithful students.

